# Development of Local Circuit Connections to Hilar Mossy Cells in the Mouse Dentate Gyrus

**DOI:** 10.1523/ENEURO.0370-18.2019

**Published:** 2019-03-26

**Authors:** Yulin Shi, Steven F. Grieco, Todd C. Holmes, Xiangmin Xu

**Affiliations:** 1 Department of Anatomy and Neurobiology, School of Medicine, University of California, Irvine, CA 92697-1275; 2 Department of Physiology and Biophysics, University of California, Irvine, CA 92697-4560; 3 Department of Biomedical Engineering, University of California, Irvine, CA 92697-2715; 4 Department of Microbiology and Molecular Genetics, University of California, Irvine, CA 92697-4025

**Keywords:** development, hippocampus, excitatory input, inhibitory input, mossy neurons, synaptic connections

## Abstract

Hilar mossy cells in the dentate gyrus (DG) shape the firing and function of the hippocampal circuit. However, the neural circuitry providing afferent input to mossy cells is incompletely understood, and little is known about the development of these inputs. Thus, we used whole-cell recording and laser scanning photostimulation (LSPS) to characterize the developmental trajectory of local excitatory and inhibitory synaptic inputs to mossy cells in the mouse hippocampus. Hilar mossy cells were targeted by visualizing non-red fluorescent cells in the dentate hilus of GAD2-Cre; Ai9 mice that expressed tdTomato in GAD+ neurons, and were confirmed by post hoc morphological characterization. Our results show that at postnatal day (P)6–P7, mossy cells received more excitatory input from neurons in the proximal CA3 versus those in the DG. In contrast, at P13–P14 and P21–P28, the largest source of excitatory input originated in DG cells, while the strength of CA3 and hilar inputs declined. A developmental trend was also evident for inhibitory inputs. Overall inhibitory input at P6–P7 was weak, while inhibitory inputs from the DG cell layer and the hilus predominated at P13–P14 and P21–P28. The strength of local DG excitation and inhibition to mossy cells peaked at P13–P14 and decreased slightly in older P21–P28 mice. Together, these data provide new detailed information on the development of local synaptic connectivity of mossy cells, and suggests mechanisms through which developmental changes in local circuit inputs to hilar mossy cells shape their physiology and vulnerability to injury during postnatal periods.

## Significance Statement

Mossy cells of the dentate gyrus (DG) have been implicated in hippocampal circuits regulating pattern separation, an important function attributed to the DG. However, physiologic inputs regulating mossy cell activity are incompletely understood. Here, we show development-dependent changes in the sources of both excitatory and inhibitory inputs. Our results suggest that excitatory inputs from the DG and local inhibitory inputs are positioned to powerfully sculpt receptive fields in mature mossy cells.

## Introduction

Mossy cells are principal excitatory neurons in the dentate gyrus (DG) of the hippocampal formation (Soriano and Frotscher, 1994; Scharfman, 1995; Scharfman and Myers, 2012). Mossy cells are of significant interest, as they are an important circuit element within the DG, which has been implicated in mediating cognitive functions such as pattern separation (Sass et al., 1992; Scharfman, 2007; Myers and Scharfman, 2011). In addition, mossy cells have been proposed to play an important role in temporal lobe epilepsy, as selective loss of DG neurons accompanies this disorder, and mossy cells appear to be among the neurons most vulnerable to injury and cell death (Sloviter, 1987). Very recently, three studies functionally characterized mossy cells, focusing particularly on *in vivo* firing properties distinguishing mossy cells from granule cells, another major neuron type in the DG, during behavior (Danielson et al., 2017; GoodSmith et al., 2017; Senzai and Buzsáki, 2017). Mossy cells fire frequently and possess multiple place fields, while granule cells exhibit extremely sparse and selective firing and the majority of these neurons possess a single place field. The new findings prompt intriguing questions regarding mossy cell circuit connections and information flow within the DG circuitry (Nakazawa, 2017a).

Anatomic circuit connections within the DG have received significant experimental attention, with many studies focusing on the DG granule cells (Amaral, 1978; Buckmaster et al., 1992, 1996; Buckmaster and Schwartzkroin, 1994; Scharfman, 2007; Scharfman and Myers, 2012; Scharfman and Bernstein, 2015). However, a detailed understanding of the excitatory and inhibitory synaptic inputs to hilar mossy cells is still lacking. Furthermore, little is known about the development of local circuit connections to mossy cells. Our recent rabies tracing work supports that mossy cells are major local circuit integrators (Sun et al., 2017), and exert feedback modulation of DG functioning. In addition, the evolution of functional circuit connections is correlated to the development of the spatial representation system in the rodent hippocampal formation (Langston et al., 2010). It is important to note that a rudimentary map of space is already present when young rat pups (2.5 weeks old) explore an open environment outside their nest for the first time; grid and place cells continue to evolve, with many grid cells not reaching adult-like formation until approximately four weeks of age (Langston et al., 2010). Thus, characterizing the development of afferent inputs to mossy cells is instrumental for understanding mossy cell place-specific firing properties and their contributions to hippocampal function.

In the present study, we use a laser scanning photostimulation (LSPS)-based approach to map and compare synaptic inputs of mossy cells across postnatal development (at ages P6–P7, P13–P14, and P21–P28). LSPS combined with whole-cell recordings has been an effective approach in elucidating cortical circuit organization, as it allows presynaptic inputs to single neurons to be mapped with high resolution glutamate-uncaging across a large anatomic area (Kuhlman et al., 2013; Sun et al., 2014; Xu et al., 2010, 2016a). Using this physiologic mapping approach, we provide a quantitative assessment of the spatial distribution and input strength of excitatory and inhibitory inputs to mossy cells across the DG and CA3 areas. Our results provide a detailed characterization of the functional organization of afferent inputs to mossy cells at different postnatal ages. These findings are relevant to understanding the *in vivo* physiology and function of mossy cells, and will advance our understanding of the role of mossy cells in both health and disease.

## Materials and Methods

### Hippocampal slice preparations

Sixty double-transgenic Ai9-tdTomato (RRID:IMSR_JAX:007905) X GAD2-ires-Cre (RRID:IMSR_JAX:010802) male and female mice were used in these experiments. All experiments were conducted in accordance with procedures approved by the Institutional Animal Care and Use Committee at the University of California, Irvine. We obtained one to three high-quality hippocampal horizontal slices from each mouse in which the DG and CA3 structures were clearly visible. To prepare living brain slices, animals of three different ages [postnatal day (P)6–P7, P13–P14, and P21–P28] were deeply anesthetized with Nembutal (>100 mg/kg, i.p.), rapidly decapitated, and their brains removed.

Hippocampal slices (400 µm thick) were cut at an angle of 20–30° to the horizontal plane to conserve intrahippocampal axonal projections (Kopanitsa et al., 2006) in well oxygenated (95% O_2_–5% CO_2_), ice-cold sucrose-containing cutting solutions (85 mM NaCl, 75 mM sucrose, 2.5 mM KCl, 25 mM glucose, 1.25 mM NaH_2_PO_4_, 4 mM MgCl_2_, 0.5 mM CaCl_2_, and 24 mM NaHCO_3_). Slices were incubated for at least 30 min in sucrose-containing ACSF at 32°C before being transferred into slice-recording chambers with standard ACSF (126 mM NaCl, 2.5 mM KCl, 26 mM NaHCO_3_, 2 mM CaCl_2_, 2 mM MgCl_2_, 1.25 mM NaH_2_PO_4_, and 10 mM glucose). Throughout cutting, incubation, and recording, ACSF was continuously supplied with 95% O_2_–5% CO_2_.

### Electrophysiology and LSPS

**Figure 1.**
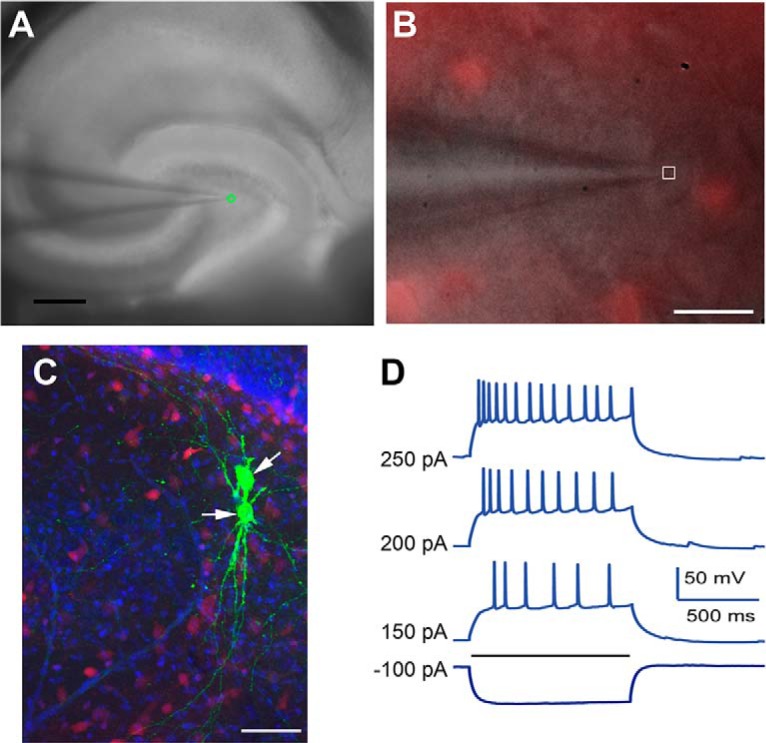
Targeted recordings of hilar mossy cells. ***A***, Horizontal hippocampal slices are acutely prepared from GAD-Cre; Ai9 tdTomato double transgenic mice and are visualized under a 4× objective. Whole-cell recordings are made from mossy cells in the DG (green circle). Scale bar = 250 μM. ***B***, Mossy cells, as shown with a 60× objective, are first identified by their lack of tdTomato fluorescence (under the pipette, white square) in the hilar region of GAD-Cre; Ai9 mice. Scale bar = 50 μM. ***C***, Morphology of recorded mossy cells (white arrows), which are injected with biocytin (green), demonstrates multipolar soma and thick dendrites with thorny excrescences. Scale bar = 50 μM. ***D***, Mossy cells have regular/adapting spiking in response to current injection (horizontal black line) through the patch pipette.

We have previously described our methods for electrophysiological recording, imaging, and photostimulation in detail, including the definitions of all reported parameters (Xu et al., 2006, 2010; for our more recent publications using these same methods see, Kuhlman et al., 2013; Sun et al., 2014; Xu et al., 2016a). Briefly, whole-cell recordings were performed in oxygenated ACSF at room temperature under a differential interference contrast (DIC)/fluorescent Olympus microscope (BX51WI). ACSF was fed into the slice recording chamber through a custom-designed flow system driven by pressurized 95% O_2_–5% CO_2_ (3 PSI) with a perfusion flow rate of ∼2 ml/min. Slices were first carefully examined under a 4× objective to target the DG using visual landmarks (Amaral, 1978). To perform whole-cell recordings, neurons were visualized at high magniﬁcation (60× objective, 0.9 NA; LUMPlanFl/IR, Olympus America Inc). Mossy cells targeted for recording were at least 50 μm below the surface of the slice and were initially identified based on the multipolar appearance of the cell soma and presence of a thick apical dendrite, and later with fluorescent imaging confirming the absence of GAD expression (Fig. 1). Patch pipettes (4- to 6-MΩ resistance) made of borosilicate glass were ﬁlled with an internal solution containing 126 mM K-gluconate, 4 mM KCl, 10 mM HEPES, 4 mM ATP-Mg, 0.3 mM GTP-Na, and 10 mM phosphocreatine (pH 7.2, 300 mOsm) when measuring EPSCs and action potentials (APs). No correction was made for the liquid junction potential. Once the glass pipette formed a gigaohm seal with the recorded cell membrane, the capacitance compensation function of the Multiclamp 700B was used for automatic compensations of cell membrane capacitance (Cm). In separate experiments, a cesium-based internal solution containing 130 mM CsOH, 130 mM D-gluconic acid, 0.2 mM EGTA, 2 mM MgCl_2_, 6 mM CsCl, 10 mM HEPES, 2.5 mM ATP-Na, 0.5 mM GTP-Na, and 10 mM phosphocreatine (pH 7.2, 300 mOsm) was used to voltage clamp pyramidal neurons at the excitatory reversal potential (0–5 mV) and measure IPSCs. Electrodes also contained 0.1% biocytin for post hoc anatomic cell labeling. Once stable whole-cell recordings were achieved with good access resistance (usually ∼30 MΩ), basic electrophysiological properties were characterized using hyperpolarizing and depolarizing current injections. Electrophysiological data were acquired with a Multiclamp 700B amplifier (Molecular Devices), data acquisition boards (models PCI MIO 16E-4 and 6713, National Instruments), and a custom-modified version of Ephus software 5. Data were digitized at 10 kHz and stored on a computer.

During photostimulation experiments, the microscope objective was switched from 60× to 4× for LSPS. The same low-power objective lens was used for delivering ultraviolet flash stimuli. Stock solution of MNI-caged-l-glutamate (Tocris Bioscience) was added to 20 ml of circulating ACSF for a concentration of 0.2 mM caged glutamate. Hippocampal slice images were acquired using the 4× objective with a high-resolution digital CCD camera, which in turn was used for guiding and registering photostimulation sites. A laser unit (DPSS Lasers) was used to generate 355-nm UV laser pulses for glutamate uncaging. Short pulses of laser flashes (1 ms, 20 mW) were delivered using an electro-optical modulator and a mechanical shutter. The laser beam formed uncaging spots, each approximating a Gaussian profile with a width of 100 μm in the focal plane.

As in previous studies, whole-cell recording of a single neuron was accompanied by laser stimulation at nearby sites, generating APs from neurons in targeted areas via LSPS-guided glutamate uncaging (Kuhlman et al., 2013; Sun et al., 2016; Xu et al., 2016a). Voltage clamping the recorded neuron allowed determination of sites contributing synaptic input. By systematically surveying synaptic inputs from hundreds of different sites across a large region, aggregate synaptic input maps were generated for individual neurons. For our mapping experiments, a standard stimulus grid (12 × 12 stimulation sites covering an area of 500 × 600 µm) was used to survey synaptic input arising from hippocampal regions of interest, including the DG, hilus, CA3, and CA1. The LSPS site spacing was empirically determined to separate adjacent stimulation sites by the smallest predicted distance in which photostimulation differentially activated adjacent neurons. Glutamate uncaging laser pulses were delivered sequentially in a nonraster, nonrandom sequence, following a “shifting-X” pattern designed to avoid revisiting the vicinity of recently stimulated sites (Shepherd et al., 2003). Because glutamate uncaging agnostically activates both excitatory and inhibitory neurons, we empirically determined the excitatory and inhibitory reversal potentials in mossy cells to isolate EPSCs and IPSCs. We voltage clamped the targeted cells at −70 mV to detect EPSCs, and use the holding potential (0–5 mV) for IPSC detection with the cesium-containing internal solution.

Photostimulation data analysis has been described in detail (Shi et al., 2010; Sun et al., 2016). Responses occurring within the 10‐ms window from laser onset are considered direct. Synaptic currents with such short latencies are not possible because they occur before the generation of APs in photostimulated neurons. Therefore, direct responses are excluded from synaptic input analysis. It is possible that smaller synaptic responses could be masked by direct glutamate responses. However, at some locations, synaptic responses were over‐riding on the relatively small direct responses and were identified and included in synaptic input analysis. The input value of each stimulation site was measured by the temporal summation (i.e., area under a curve) of individual EPSCs or IPSCs from each photostimulation site with the baseline spontaneous response subtracted, and then normalized by the analysis window of 150 ms after photostimulation. While the value actually represents synaptic charge, for consistency with previous studies and because synaptic current is a more familiar unit, this average integrated value is expressed in picoamperes. As for individual map construction, input measurements from different stimulation sites were assigned to their corresponding anatomic locations in the hippocampus; color‐coded maps of average input amplitude and the number of events per site were plotted to illustrate overall input patterns to the recorded cell.

To quantitatively compare input strengths and patterns across cell groups, we summed the ESPC/IPSC input amplitudes and the numbers of EPSCs/IPSCs within and across specific hippocampal subfields for individual cells. These measurements are termed the summed or total input (excitation/inhibition) and the summed or total numbers of EPSCs/IPSCs of each cell. We also performed analysis of EPSC/IPSC characteristics including their rise time, time constant, and onset latency.

### Morphologic examination and cell-type identification

After electrophysiological recording, brain slices were fixed in 4% paraformaldehyde overnight, then transferred to 30% sucrose solution in PBS. Slices were stained against biocytin with 1:500 Alexa Fluor 488-conjugated streptavidin (Jackson ImmunoResearch) to visualize the morphology of recorded cells. Slices were also stained for 4′-6-diamidino-2-phenylindole (DAPI; Sigma-Aldrich) to identify laminar boundaries. Cell morphology was visualized using Olympus BX 61 epifluorescent microscopy and the MetaMorph imaging suite (Molecular Devices). In addition, we imaged labeled cells in selected sections with a confocal microscope (LSM 700/780, Carl Zeiss Microscopy). Image stitching, overlaying, maximum projections, and export were performed by using the ZEN software analysis tools.

### Experimental design and statistical analysis

Data are reported as mean values ± SEM. All statistical analyses were performed using GraphPad Prism version 7.00 for Windows (GraphPad Software). For statistical comparisons between groups, data were checked for normality and equal variance. For statistical comparisons across the three developmental ages, we used the Kruskal–Wallis test (a non-parametric one-way ANOVA) and Mann–Whitney *U* tests or one-way ANOVAs with Tukey’s *post hoc* tests were used for group comparisons. In all experiments, the level of statistical significance was defined as *p* < 0.05.

## Results

### Development changes in intrinsic physiologic properties of hilar mossy cells

Currently, there are no transgenic methods for directly distinguishing mossy cells from other hilar neurons. Therefore, we use GAD2-Cre; Ai9 mice to facilitate mossy cell identification and recording. In these mice, non-mossy cell inhibitory neurons (GAD-expressing) are fluorescently labeled. Using a 60× objective, excitatory mossy cells are targeted for recording. These neurons appeared as shadowed cell bodies, surrounded by fluorescently labeled GAD-expressing inhibitory neurons (Fig. 1*B*). Biocytin infusion during whole-cell recording and *post hoc* staining was used to identify all mossy cells based on morphology (Fig. 1*C*). The identity of mossy cells could be verified by their characteristic regular-adapting spiking patterns in response to suprathreshold current injection when the K^+^ containing internal solution was used for electrical recordings (Fig. 1*D*).

**Figure 2.**
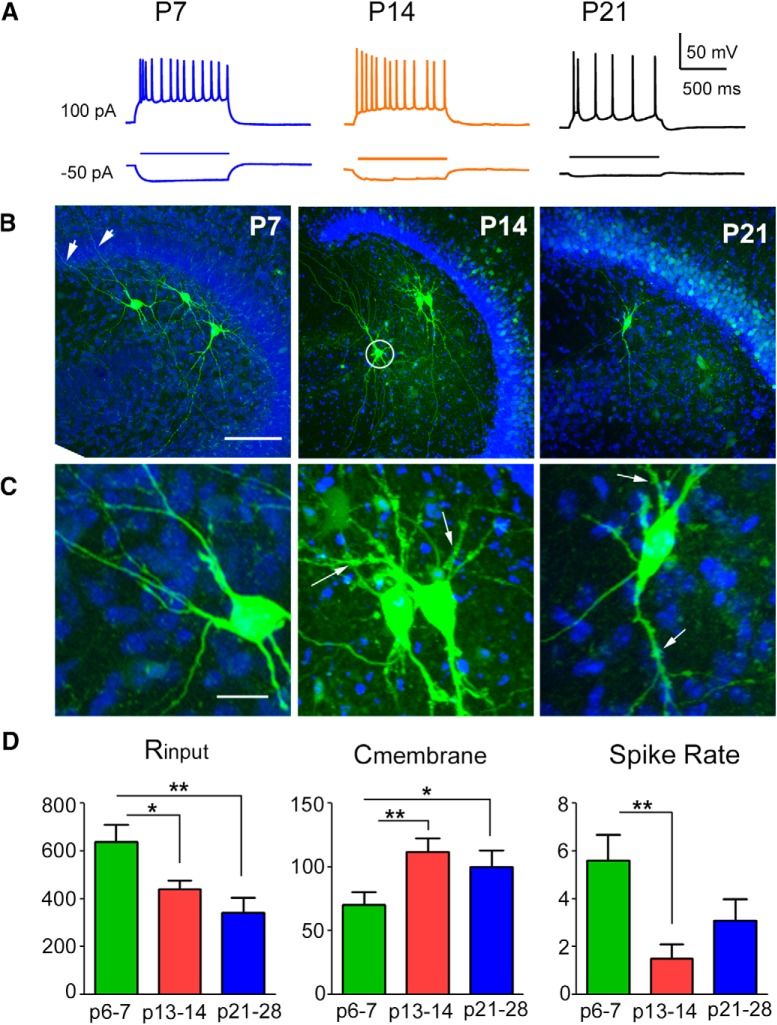
Developmental changes in intrinsic physiologic and morphologic properties of mossy cells. ***A***, Example responses to current injection (horizontal lines) in example mossy cells recorded from P7, P14, and P21 mice. ***B***, ***C***, Example morphology of biocytin-labeled mossy neurons (green) and surrounding DAPI-stained tissue (blue) at P7, P14, and P21. The labeled neurons in the P7 mouse had proximal dendrites with branches penetrated into the fascia dentate (arrows for P7 mouse in ***B***) and were relatively smooth (image shown in ***C***), while neurons recorded in P14 and P21 mice have obvious thorny excrescence (arrows in ***C***) on proximal dendrites. Scale bar = 250 μM (***B***) and 25 μM (***C***). All labeled neurons had large, multipolar somata, and thick thorny proximal dendrites. A neuron (circle in ***B***) in the P14 mouse is in CA3. ***D***, The input resistance (left, measured in MΩ) decreased with age from P6–P7 to P13–P14, and P21–P28. The Cm (middle, measured in pF), is the capacitance of the cell membrane and increased with age from P6–P7 to P13–P14, and P21–P28. The spike rate (right, measured in Hz) is the number of evoked spikes in response to current injection of 100 pA during recording, and decreased with age from P6–P7 to P13–P14. * indicates the statistical significance (*p* < 0.05), and ** indicates *p* < 0.01. Also see Table 2.

Intrinsic physiologic properties of each recorded neuron were acquired immediately after successful break-in with the recording electrode containing the K^+^ internal solution. Significant differences in physiologic property measures were found in comparing the three developmental age groups studied (P6–P7, P13–P14, P21–P28; Fig. 2*A*,*D*). Input resistance, measured with the membrane voltage change by hyperpolarizing current injection during whole-cell recording (Rinput, in the unit of MΩ) declined with increasing age (Table 1). The Rinput of P6–P7 mossy cells was significantly larger than that of P13–P14 and P21–P28 neurons (*p* = 0.02 and *p* = 0.008, respectively). The average Rinput of P6–P7 mossy cells (*N* = 11) was 637.7 ± 74 versus 439.8 ± 37.7 in P13–P14 mossy cells (*N* = 12) and 341.7 ± 63.5 in P21–P28 (*N* = 13) mossy cells. Monotonically declining Rinput values could result from increased conductance of ion channels, or increased ion channel number in the cell membrane. Cm increased in older animals, with significantly lower values present in the P6–P7 group (*p* = 0.02 and *p* = 0.008 vs P13–P14 and P21–P28, respectively; Table 1). However, resting membrane potential (RMP) did not change significantly across developmental ages.

**Table 1.** Intrinsic physiologic properties of mossy cells at different ages

Mouse age	RMP (mV)	Rs (MΩ)	Rinput (MΩ)	Cm (pF)	Evoked spike rate (Hz)
P6–P7 (*N* = 11)	–60.3 ± 1.7	32.6 ± 2.4	637.7 ± 74.0	70.4 ± 10.3	5.6 ± 1.1
P13–P14 (*N* = 12)	–61.5 ± 1.1	27.2 ± 3.0	439.8 ± 37.7	111.8 ± 10.7	1.5 ± 0.6
P21–P28 (*N* = 13)	–62.7 ± 1.6	33.4 ± 3	341.7 ± 63.5	99.9 ± 13	3.1 ± 0.9
*p* value	ns	ns	*P6–P7 vs P13–P14, 0.02 *P6–P7 vs P21–P28, 0.008	*P6–P7 vs P13–P14, 0.02 *P6–P7 vs P21–P28, 0.008	*P6–P7 vs P13–P14, 0.006

RMP, resting membrane potential; Rs, access resistance; Cm, membrane capacity; Rinput, input resistance. ns, not significant for statistical comparison. * indicate the statistical significance of *p* < 0.05.

**Table 2.** Statistics of LSPS-mapped EPSC inputs to hilar mossy cells at different ages

		P6–P7	P13–P14	P21–P28	Significance level
DG	Photostimulation evoked input (EI)	44.8 ± 8.4	189.9 ± 59.0	105.9 ± 12.4	*P6–P7 vs P13–P14, 0.05 *P6–P7 vs P21–P28, 0.0005
	EI per site	3.6 ± 0.5	9.6 ± 1.5	8.3 ± 1.4	ns
	% EI	29.1 ± 6.1	62.7 ± 6.2	72.1 ± 8.4	ns
hilus	EI	18.8 ± 4.6	10.9 ± 6.6	5.3 ± 2.6	*P6–P7 vs P21–P28, 0.022
	EI per site	4.1 ± 1.0	3.1 ± 1.3	1.4 ± 0.4	ns
	% EI	18.2 ± 6.3	4.8 ± 1.7	3.6 ± 1.8	ns
CA3	EI	73.1± 19.9	43.7 ± 14.6	20.7 ± 7.9	*P6–P7 vs P21–P28, 0.0056
	EI per site	3.7 ± 0.5	3.9 ± 0.7	2.5 ± 6.3	ns
	% EI	32.0 ± 5.4	22.4 ± 4.6	14.0 ± 5.3	ns

Note that the recorded cells used for this table include 12 cells (P6–P7), 9 cells (P13–P14), and 10 cells (P21–P28). EI: photostimulation-evoked postsynaptic input measured from recorded neurons; %EI: the regional percentage of total evoked input; EI per site: evoked input per photostimulation. * indicates the statistical significance of *p* < 0.05.

After recording, neurons were immunostained with biocytin, and cell morphology was recovered using confocal imaging (Fig. 2*B*,*C*). Young neurons (P6–P7) had relatively smooth proximal dendrites, while dendrites in older animals (P13–P14 and P21–P28) had many thorny excrescences that were confined to the hilus. These morphologic changes are consistent with changes in the Cm that is proportional to the cell surface area. Compared to smooth dendritic surfaces of mossy cells in young ages, the larger thorny surfaces at older ages are correlated to larger cell Cm (see above). Further, thorny excrescences are clusters of complex spines on proximal dendrites of mossy cells, which indicate stronger synaptic connections between dentate granule cells and their postsynaptic target neurons in older age groups. Thus, these morphologic changes are expected to be correlated with stronger excitatory inputs from dentate granule cells to mossy cells in older ages (see below).

### LSPS responses

**Figure 3.**
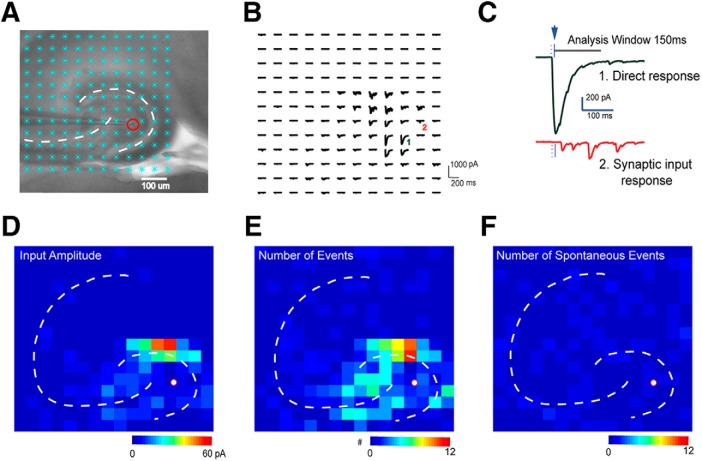
LSPS mapping and data analysis. ***A***, A horizontal hippocampal slice under a 4× objective with a patched neuron (red circle) and laser stimulation sites overlaid (cyan asterisks). ***B***, Raw signal traces recorded from the patched neuron during laser stimulation. ***C***, Examples of a direct response (top green trace) which has a large amplitude and a short response latency, and synaptic responses (bottom red) which have smaller amplitudes and longer latencies. ***D–F***, Synaptic responses were detected and extracted using automatic software processing (Shi et al., 2010). Input amplitude (see Methods) (***D***), the number of evoked synaptic events, as defined by the number of EPSCs elicited per laser pulse (***E***), and the number of spontaneous events (***F***) were plotted in heat maps.

LSPS can induce two major forms of responses: (1) direct activation of the recorded neuron’s glutamate receptors after glutamate uncaging; and (2) synaptically-mediated EPSCs or IPSCs resulting from suprathreshold activation of presynaptic neurons. Excitatory responses occurring within 10 ms after laser onset are caused by direct activation and exhibited larger amplitudes and a distinct shape (longer rise time) occurring at short latencies after laser stimulation (Fig. 3*A–C*). Direct responses are excluded from local synaptic input analysis. However, at some locations, synaptic responses are superimposed on relatively small direct responses and were included in synaptic input analysis.

After each LSPS trial, we obtain a map of the raw signal traces (Fig. 3*B*). A short detection window (10–160 ms after stimulation) is used to reduce the probability of detecting spontaneous and polysynaptic events. Custom-written software (Shi et al., 2010) is used to isolate the synaptically-mediated responses from direct responses and to calculate the average amplitude, integrated input, event number, first event delay, rise time, and decay constant of each synaptic response. These quantitative data are turned into color-coded maps for further region-specific analysis (Fig. 3*D–F*). To reduce contamination by spontaneous events, we average results from multiple photostimulation trials, and only anatomic sites with EPSCs or IPSCs in all experiments are identified as presynaptic inputs. This temporally precise approach shows that LSPS can be used for local circuit mapping of monosynaptic excitatory and inhibitory connections to hilar mossy cells.

### Spatial precision of LSPS

**Figure 4.**
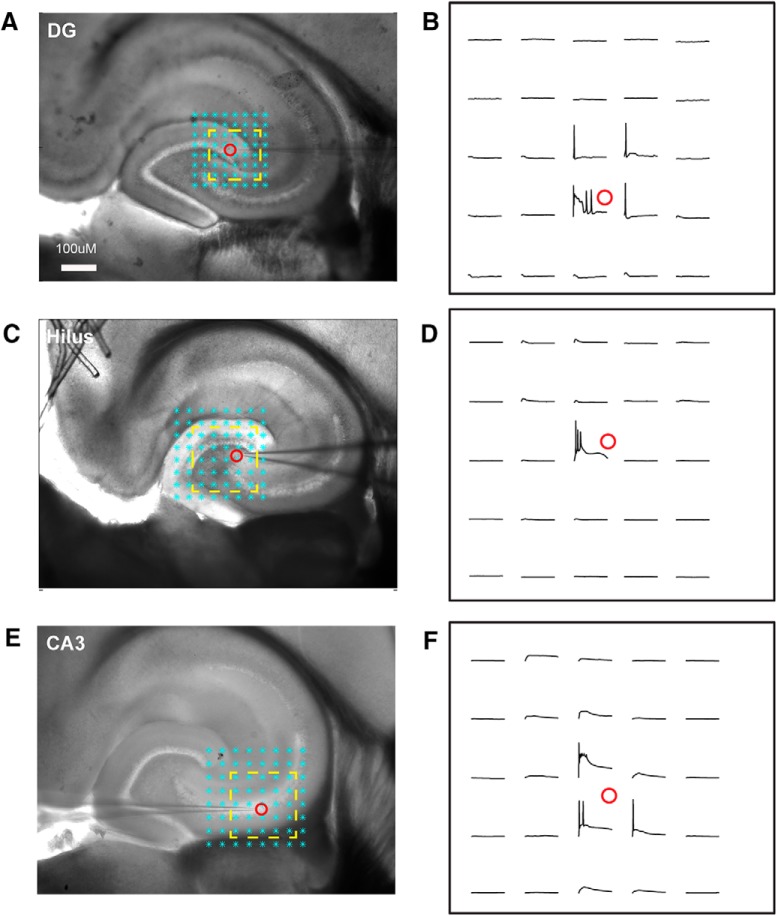
Spatial precision of LSPS mapping for neurons in the DG, hilus, and CA3c. ***A***, ***B***, Excitation profile of a recorded DG granule cell from a mouse hippocampal slice. The excitation profile shows the spatial distribution of uncaging sites that produce APs. The cell was held in current clamp mode. The cyan asterisks in ***A*** are the stimulation sites (75-µm spacing). The evoked APs were restricted to a small region (yellow square). Raw signal traces within the yellow square are shown in ***B***. ***C***, ***D***, Excitation profile of a hilar mossy cell from a mouse hippocampal slice (100-µm spacing). ***E***, ***F***, Excitation profile of CA3 pyramid cells from mouse hippocampal slice (100-µm spacing). Excitation profiles show no spike-evoking sites distal from the perisomatic area of the recorded neuron, demonstrating that LSPS maps (e.g., Figures 5 and 6) represent input from monosynaptic connections.

Before mapping synaptic inputs, we also characterized the spatial extent over which neurons within the photostimulation area respond to laser pulses. We recorded from neurons in the current clamp mode and examined APs elicited by photostimulating in the area around the recorded neuron (Fig. 4). The stimulation pattern is typically an 8 × 8 grid centered on the recorded neuron, with width and length dimensions that ranged from 75 to 100 µm, depending on the age of the slice. Generally, photostimulation proximal to the clamped neuron is required to elicit APs. Across the developmental ages studied, the average distance of photostimulation-evoked spikes from recorded cell bodies was 115.7 ± 12.7 μm (*N* = 7 slices), 133.3 ± 26.7 μm (*N* = 3), and 127.5 ± 8.9 μm (*N* = 12), for DG granule cells, hilar neurons, and proximal CA3 cells, respectively. Further, the excitability of the presynaptic sources that is measured by the average number of evoked spikes per cells did not differ significantly across the age groups. These data show that LSPS can be used for local circuit mapping of monosynaptic excitatory and inhibitory connections to hilar mossy cells.

### Excitatory synaptic inputs to mossy cells

**Figure 5.**
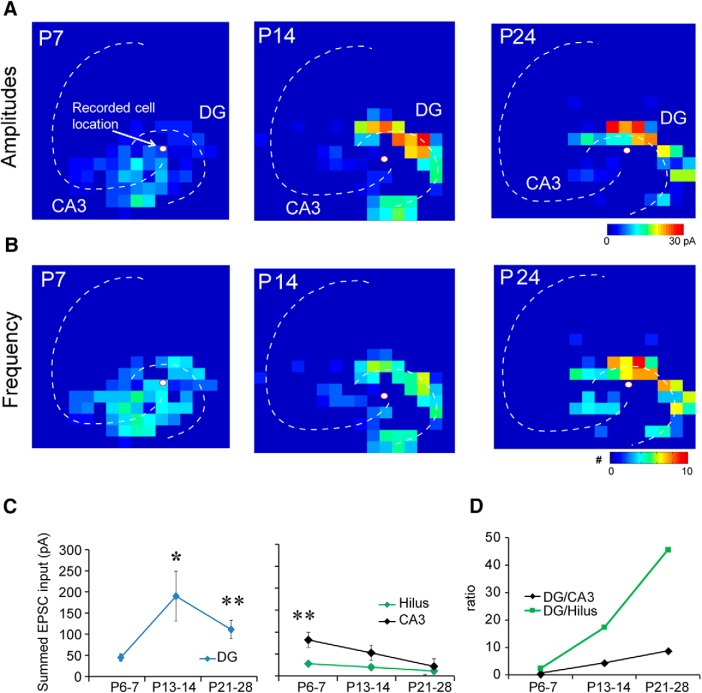
Functional maps of excitatory inputs to mossy cells across developmental ages. ***A***, Strength of excitatory input (input amplitudes) at P7, P14, and P24 for a representative mossy cell. ***B***, Frequency of EPSC events (number of evoked synaptic events per second) for a representative mossy cell. ***C***, ***D***, Summed (averaged) amplitude of excitatory inputs to mossy cells. We recorded from 12, 9, and 10 cells from P6–P7, P13–P14, and P21–P28 mice, respectively. In the left two panels in ***C***, the *y*-axis indicates input strength. In the right panel in ***D***, the *y*-axis shows the ratio of input strengths. * indicates the statistical significance (*p* < 0.05), and ** indicates *p* < 0.01. Also see Table 2.

A total of 31 mossy cells were recorded from hippocampal slices taken from P6–P7, P13–P14, and P21–P28 mice (*N* = 12, *N* = 9, and *N* = 10 neurons, respectively; Fig. 5). Their average amplitude of summed excitatory inputs (Shi et al., 2010; Sun et al., 2014; Xu et al., 2016b) from the DG was weak in P6–P7 mice, and significantly increased in P13–P14 and P21–P28 mice (Fig. 5*A*,*C*,*D*). Although the excitation from the DG declined slightly at P21–P28, this was not a statistically significant change (vs P13–P14 mice). We found that mossy cells at P6–P7 receive a majority of their excitatory inputs from the CA3 (Fig. 5*C*, right panel, *D*), indicating the presence of a strong CA3 backprojection. As development proceeded, the strength of CA3 and hilar inputs diminished and mossy cells received more of their excitatory input from the DG (Fig. 5*C*,*D*). Consistent with summed input measurements, recorded cells received average integrated excitatory input of 44.8 ± 8.4 pA from the DG at P6–P7; 189.9 ± 59.0 pA at P13–P14; and 105.9 ± 12.4 pA at P21–P28. The DG input constituted 29.1 ± 6.1% of the total excitation received by mossy cells at P6–P7, 62.7 ± 6.2% at P13–P14, and 72.2 ± 7.1% at P21–P28. The input per stimulation site was 3.6 ± 0.5 pA at P6–P7 to 9.6 ± 1.5 and 8.3 ± 1.3 pA at P13–P14 and P21–P28, respectively (Table 2).

Relative to the input from the DG, the CA3 provided more input at P6–P7 and much less input at P13–P14 and P21–P28 (Fig. 5*C*,*D*). The integrated input from CA3 was 73.1 ± 19.9 pA at P6–P7, 43.7 ± 14.6 pA at P13–P14, and 20.7 ± 7.9 pA at P21–P28. Input from the hilus followed a similar pattern. The integrated input from the hilus was 18.8 ± 4.6 pA at P6–P7, 10.9 ± 6.6 pA at P13–P14, and 5.3 ± 2.5 pA at P21–P28. The average percentage of evoked excitatory inputs from the hilus was 18.2 ± 6.3%, 4.8 ± 1.7%, and 3.6 ± 1.7% for P6–P7, P13–P14, and P21–P28, respectively. The per-stimulation site input decreased from 4.1 ± 1.0 pA at P7 to 3.1 ± 1.3 pA and 1.4 ± 0.5 pA at P13–P14 and P21–P28, respectively (Table 2).

The event frequency, which represents the number of evoked synaptic events per second to a recorded neuron, was also measured (Fig. 5*B*). The number of EPSCs evoked per DG stimulation site was 3.2 ± 0.5, 3.9 ± 0.4, and 3.4 ± 0.1 in P6–P7, P13–P14, and P21–P28 mice, respectively. The number of evoked EPSCs per hilar stimulation site was 3.3 ± 0.6, 1.7 ± 0.5, and 0 ± 00 in P6–P7, P13–P14, and P21–P28 groups, respectively. The number of EPSCs evoked per CA3 stimulation site was 3.4 ± 0.4, 2.4 ± 0.3, and 1.7 ± 0.3 in P6–P7, P13–P14, and P21–P28 groups, respectively. The average latencies of EPSCs did not differ significantly between the three age groups (Table 3). We examined the rise time, time constant and onset latency of EPSCs mapped from DG, hilus and CA3 (Table 4). We did not find age-related differences except the EPSC rinse time difference of CA3 inputs.

**Table 3.** Number of EPSCs recorded from mossy cells at different ages

		P6–P7	P13–P14	P21–P28	Significance level
DG	Total numbers	38.9 ± 7.0	70.7 ± 21.2	50.3 ± 9.3	ns
	Numbers per site	3.2 ± 0.5	3.9 ± 0.4	3.4 ± 0.1	ns
	% Total events	31.2 ± 5.0	56.6 ± 6.4	56.7 ± 10.4	ns
Hilus	Total numbers	15.1 ± 3.4	4.9 ± 2.5	5.4 ± 2.7	*P6–P7 vs P13–P14, 0.05 *P6–P7 vs P21–P28, 0.0117
	Numbers per site	3.3 ± 0.6	1.7 ± 0.5	0.0	ns
	% Total events	13.3 ± 4.3	5.4 ± 2.1	6.0 ± 3.0	ns
CA3	Total numbers	60.4 ± 13.9	25.5 ± 8.4	20.5 ± 9.1	*P6–P7 vs P21–P28 0.0193
	Numbers per site	3.4 ± 0.4	2.4 ± 0.3	1.7 ± 0.3	ns
	% Total events	33.4 ± 5.8	26.2 ± 4.7	23.1 ± 10.9	ns

* indicates the statistical significance of *p* < 0.05.

**Table 4.** Rise time, time constant, and onset latency of EPSCs

		P6–P7	P13–P14	P21–P28	Significance level
DG	Rise time (ms)	2.4 ± 0.12	2.6 ± 1.3	2.5 ± 0.1	ns
	Time constant (ms)	4.2 ± 0.2	4.2± 0.3	5.0 ± 0.6	ns
	Latency (ms)	50.5 ± 5.6	46.2 ± 6.7	42.1 ± 4.8	ns
Hilus	Rise time (ms)	2.3 ± 0.2	2.9 ± 0.6	2.2 ± 0.2	ns
	Time constant (ms)	6.7 ± 1.0	5.7 ± 0.5	6.2 ± 1.5	ns
	Latency (ms)	46.9 ± 4.9	59.1 ± 7.3	39.2 ± 9.1	ns
CA3	Rise time (ms)	2.4 ± 0.1	3.1 ± 0.1	2.7 ± 0.3	*P6–P7 vs P13–P14, 0.004 *P13–P14 vs P21–P28, 0.04
	Time constant (ms)	7.2 ± 0.3	4.4 ± 0.3	3.4 ± 0.9	ns
	Latency (ms)	44.1 ± 3.0	51.0 ± 7.4	47.2 ± 10.3	ns

* indicates the statistical significance of *p* < 0.05.

We calculated the number of input locations activated as a DG/CA3 connectivity ratio and found that the ratio for EPSCs trends very strongly (*p* = 0.0539; Kruskal–Wallis test) toward increasing from P6–P7 to P21–P28 supporting our hypothesis that excitatory inputs shift to the DG by the end of development. We also analyzed the distances of recorded mossy cells to the DG and CA3 and confirmed that recording location differences between age groups did not account for our results.

### Inhibitory synaptic inputs to mossy cells

**Figure 6.**
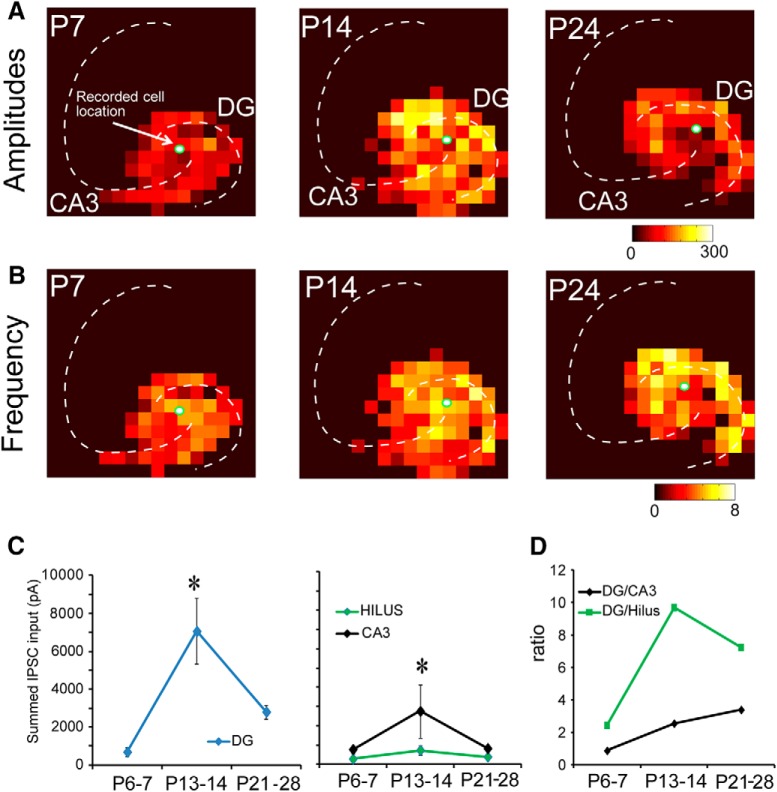
Functional maps of inhibitory inputs to mossy cells across developmental ages. ***A***, Strength of inhibitory input amplitude at P7, P14, and P24 for representative mossy cells. ***B***, Frequency of IPSC events (number of evoked synaptic events per second) for representative mossy cells. ***C***, ***D***, Summed (averaged) amplitude of inhibitory inputs to mossy cells. We mapped 10, 7, and 16 cells from P6–P7, P13–P14, and P21–P28 mice, respectively. In the left two panels in ***C***, the *y*-axis indicates input strength. In the right panel in ***D***, the *y*-axis shows the ratio of input strengths. * indicates the statistical significance (*p* < 0.05). Also see Table 5.

A total of 33 mossy cells were recorded from P6–P7, P13–P14, and P21–P28 mice (*N* = 10, *N* = 7, and *N* = 16; Fig. 6). Based on region specific-analysis of inhibitory input sources, we found that at P6–P7, mossy cells received roughly equivalent inhibitory input from the DG, hilus, and CA3 (Fig. 6*A*,*C*). As mice aged, inhibition from the DG and CA3 peaked at P13–P14 and then decreased at older ages (P21–P28; Fig. 6*C*,*D*). The amplitude of inhibitory input from the DG was significantly larger than that from the CA3. The magnitude of hilar inhibition did not significantly change over the course of development. Mossy cells received 68.5 ± 24.0 pA of integrated inhibitory input from the DG at P6–P7, 490.7 ± 100.6 pA at P13–P14, and 195.7 ± 37.0 pA at P21–P28. DG input constituted 31.2 ± 6.1% of excitatory input at P6–P7, 62.7 ± 6.2% at P13–P14, and 64.9 ± 3.5% at P21–P28. The magnitude of inhibitory input per stimulation site was 3.9 ± 0.9, 12.8 ± 1.4, and 6.6 ± 0.8 pA at P6–P7, P13–P14, and P21–P28, respectively (Table 5). The CA3 provided more inhibition at P13–P14 and substantially less input at P6–P7 and P21–P28. Integrated inhibitory input from CA3 neurons was 77.1 ± 20.5 pA at P6–P7, 150.6 ± 30.4 pA at P13–P14, and 51.7 ± 13.5 pA at P21–P28. CA3 neurons provided 40.5 ± 6.2%, 18.5 ± 2.5%, and 11.6 ± 2.9% of inhibitory input to mossy cells at P6–P7, P13–P14, and P21–P28, respectively. Average inhibitory input per stimulation site was 4.3 ± 0.8, 10.8 ± 2.1, and 4.2 ± 0.5 pA at P6–P7, P13–P14, and P21–P28 (Table 5).

**Table 5.** Statistics of LSPS-mapped IPSC inputs to hilar mossy cells at different ages

		P6–P7	P13–P14	P21–P28	Significance level
DG	Photostimulation evoked input (EI)	68.5 ± 24.0	490.7 ± 100.6	195.7 ± 37.0	*P6–P7 vs P13–P14, 0.005 *P6–P7 vs P21–P28, 0.0001
	EI per site	3.9 ± 0.9	12.8 ± 1.4	6.6 ± 0.8	ns
	% EI	31.2 ± 5.5	62.3 ± 5.0	64.8 ± 3.6	ns
Hilus	EI	28.1 ± 8.8	132.2 ± 35.2	31.6 ± 4.8	*P6–P7 vs P13–P14, 0.002 *P13–P14 vs P21–P28 0.004
	EI per site	4.7 ± 1.3	10.7 ± 2.1	4.7 ± 0.50	ns
	% EI	18.2 ± 3.9	14.9 ± 3.0	17.9 ± 4.5	ns
CA3	EI	77.1 ± 20.5	150.6 ± 30.4	51.8 ± 13.6	*P6–P7 vs P13–P14, 0.05 *P13–P14 vs P1–P28 0.002
	EI per site	4.3 ± 0.8	10.8 ± 2.1	4.2 ± 0.9	ns
	% EI	40.5 ± 6.2	18.5 ± 2.5	11.7 ± 2.9	ns

Note that the recorded cells used for this table include 10 cells (P6–P7), 7 cells (P13–P14), and 16 cells (P21–P28). EI, EI per site, and % EI are the same as in Table 2. * indicates the statistical significance of *p* < 0.05.

Inhibitory input from the hilus was generally weak and diffuse across all ages studied. Average integrated input was 28.1 ± 8.8 pA at P6–P7, 132.2 ± 35.2 pA at P13–P14, and 31.5 ± 4.7 pA at P21–P28. Input from the hilus accounted for 18.2 ± 3.9%, 14.9 ± 2.9%, and 17.8 ± 4.5% of the inhibitory input to mossy cells. Average input per stimulation site was 4.7 ± 1.3, 10.7 ± 2.1, and 4.7 ± 0.5 pA at P6–P7, P13–P14, and P21–P28, respectively (Table 5).

The frequency of LSPS-evoked IPSCs was also measured independently from IPSC strength (Fig. 6*B*). The number of evoked IPSCs per stimulation site in the DG was 2.0 ± 0.4, 3.7 ± 0.4, and 2.9 ± 0.2 at P6–P7, P13–P14, and P21–P28. The number of evoked IPSCs per stimulation site in the hilus was 2.6 ± 0.6, 3.4 ± 0.6, and 2.6 ± 0.2 in P6–P7, P13–P14, and P21–P28 mice, respectively. Finally, the number of evoked IPSCs per stimulation site in the CA3 was 2.2 ± 0.4, 3.0 ± 0.5, and 1.8 ± 0.3 in P6–P7, P13–P14, and P21–P28 mice (Table 6).

**Table 6.** Number of IPSCs recorded from mossy cells at different ages

		P6–P7	P13–P14	P21–P28	Significance level
DG	Total Numbers	32.6 ± 11.7	105.3 ± 21.1	80.4 ± 14.3	*P6–P7 vs P13–P14, 0.01 *P6–P7 vs P21–P28, 0.003
	Numbers per site	2.0 ± 0.4	3.7 ± 0.4	2.9 ± 0.2	ns
	% Total events	31.1 ± 5.6	56.3 ± 4.8	63.1 ± 3.2	ns
Hilus	Total numbers	15.2 ±4.2	35.4 ± 9.8	17.2 ± 2.4	*P6–P7 vs P13–P14, 0.05 *P13–P14 vs P21–P28, 0.02
	Numbers per site	2.6 ± 0.6	3.4 ± 0.6	2.6 ± 0.2	ns
	% Total events	19.3 ± 3.9	17.1 ± 2.6	19.1 ± 4.0	ns
CA3	Total numbers	36.2 ± 9.1	44.3 ± 13.2	21.4 ± 4.9	ns
	Numbers per site	2.2 ± 0.36	3.0 ± 0.5	1.8 ± 0.3	ns
	% Total events	39.3 ± 6.4	19.9 ± 2.7	11.9 ± 2.8	ns

* indicates the statistical significance of *p* < 0.05.

There was a general trend for the latency of IPSC onset after photostimulation to decrease from P6–P7 to P13–P14, and increase from P13–P14 to P21–P28. For DG-evoked IPSCs, the latency to IPSC onset was significantly longer at P6–P7 versus other ages, while the latency to IPSC onset in P13–P14 mice was significantly shorter than in P21–P28 animals. For CA3-evoked IPSCs, the latency to IPSC onset was significantly longer in P6–P7 mice versus other ages. Differences in the IPSC onset latency after hilar stimulation did not reach significance (Table 7).

**Table 7.** Rise time, time constant, and onset latency of IPSCs

		P6–P7	P13–P14	P21–P28	Significance level
DG	Rise time (ms)	4.1 ± 0.2	4.0 ± 0.2	4.4 ± 0.2	ns
	Time constant (ms)	9.3 ± 0.1	7.7 ± 0.8	7.4 ± 0.3	*P6–P7 vs P21–P28, 0.02
	Latency (ms)	57.1 ± 2.2	34.8 ± 1.6	40.2 ± 2.5	*P6–P7 vs P13–P14, 0.0003 *P6–P7 vs P21–P28, 0.00009 P13–P14 vs P21–P28, 0.07
Hilus	Rise time (ms)	4.6 ± 0.3	4.4 ± 0.3	4.8 ± 0.2	ns
	Time constant (ms)	7.3 ± 0.0	7.7 ± 1.0	9.4 ± 0.8	ns
	Latency (ms)	39.3 ± 6.5	28.7 ± 4.3	33.0 ± 2.9	ns
CA3	Rise time (ms)	4.4 ± 0.3	3.5 ± 0.4	4.5 ± 0.2	*P6–P7 vs P13–P14, 0.05 *P13–P14 vs P21–P28, 0.03
	Time constant (ms)	8.8 ± 0.1	7.0 ± 0.8	8.6 ± 0.6	ns
	Latency (ms)	61.5 ± 6.2	34.0 ± 4.5	35.8 ± 2.5	*P6–P7 vs P13–P14, 0.008 *P6–P7 vs P21–P28, 0.003

* indicates the statistical significance of *p* < 0.05.

We calculated the number of input locations activated as a DG/CA3 connectivity ratio. The DG/CA3 input number ratio for IPSCs increases from P6–P7 to P21–P28, also supporting our hypothesis that inhibitory inputs to mossy cells shift to the DG by the end of development as well. We also analyzed the distances of recorded mossy cells to the DG and CA3 to confirm that recording location differences between age groups did not account for our results. A difference was only observed for calculated distances of IPSC map sites in CA3 for P6–P7 versus P21–P28.

## Discussion

In the present study, LSPS combined with whole-cell patch clamping was used to assess excitatory and inhibitory synaptic inputs to mossy cells over the course of postnatal development in mice. Our study is the first to provide a comprehensive evaluation of the spatial distribution and input strength of local circuit connections to mossy cells. Both excitatory and inhibitory input to hilar mossy cells evolved dynamically over the course of development. At P6–P7, the majority of excitatory input to mossy cells came from CA3, with summed ESPC input that nearly doubled that of input from the DG. Input from the hilus was modest. Within a week (at P13–P14), input from the DG had substantially increased (nearly a four-fold increase), while excitatory inputs from both the CA3 and the hilus declined. Input from the DG remained the strongest source of excitatory drive to mossy cells at P21–P28.

A roughly similar pattern was apparent in the development of inhibitory input to mossy cells. At the youngest postnatal age studied (P6–P7), input from the CA3 predominated, with less robust input from the DG. By P13–P14, the relative strength of inhibitory input from these sources reversed, with the strongest input from the DG. Inhibitory input from the hilus was modest at all ages studied. For both excitatory and inhibitory drive, summed input strength was relatively weak at early postnatal ages, peaked at P13–P14, and declined at P21–P28.

### Multiple inputs shape mossy cell physiology

Within the DG, mossy cells, granule cells, and local interneurons display a complex pattern of recurrent connectivity that has been implicated in pattern separation, an important role of the DG. Recent studies have shown that the electrophysiological characteristics of mossy cells differ greatly from those of granule cells (Danielson et al., 2017; GoodSmith et al., 2017; Senzai and Buzsáki, 2017). While granule cells fire sparsely (at low frequency) and selectively and typically have only a single place field, mossy cells fire at higher frequencies and more promiscuously, with multiple place fields. Moreover, mossy cells remap place fields more rapidly when exposed to new environmental cues. Though mossy cells receive strong, monosynaptic excitatory drive directly from granule cells, these studies found that mossy cell firing was only rarely driven by granule cells with overlapping place fields, suggesting excitatory drive creating place fields in mossy cells likely stems from other sources. The sources of this input, important for understanding circuits underlying pattern separation, remains undefined. CA3 pyramidal cells and dentate semilunar granule cells have been proposed as candidate sources of input (Nakazawa, 2017b). There are reports of place field representations developing into adulthood in hippocampus as well (Langston et al., 2010). Indeed, while additional studies are needed to fully understand the contribution of DG and CA3 neurons to mossy cell place fields, CA3 excitatory inputs likely can contribute to interesting mossy cell physiology *in vivo* as discussed above.

Our results show that while excitatory drive from CA3 provides the bulk of the excitatory input to mossy cells in very young animals (P6–P7), this is a developmentally transient state that does not persist into adulthood. In the mature hippocampal circuit (in P21–P28 mice), a majority of the excitatory drive to mossy cells originates from neurons with cell bodies in the DG. It is possible that semilunar granule neurons provide a defining excitatory input to hilar mossy cells, as semilunar neurons are glutamatergic neurons in the inner molecular layer that provide mossy cells with excitatory input creating persistent bursting activity (Larimer and Strowbridge, 2010). Further investigation is required to determine how these neurons may contribute to receptive field structure in mossy cells.

### Comparison of excitatory and inhibitory inputs to mossy cells

Our experimental results confirm the finding that mossy cells receive strong excitatory innervations from DG granule cells in the mature hippocampus. However, our results show for the first time that excitation from the DG is weak at P6–P7 (Table 2). This is consistent with the finding that thorny excrescences appear only at rather late ages (around P9) and do not become common until P14 in the rodent (Ribak et al., 1985). Young mossy cells (in P6–P7 mice) had most of their excitatory input from CA3, although the strength of this input decreased significantly at later ages. Excitatory input from the hilus, which was overall weak at all ages studied, could result from recurrent connections originating from other mossy cells.

Inhibition of mossy cells originated predominantly from synaptic inputs from the DG and CA3. At the youngest ages studied, inhibition was relatively weak overall and came from DG, hilar, and CA3 inputs. As animals aged, inhibition from the DG increased significantly, while inhibition from the CA3 and hilus declined. Both excitation and inhibition from the DG peaked at P14 and decreased slightly at P21–P28. Although further studies are necessary to reveal the mechanism of this reduction, pruning of initially exuberant synaptic connections and decreases in DG cell density could contribute to decreased mossy cell input (Seress, 1977; Sadgrove et al., 2006).

While local excitation and inhibition to mossy cells showed some different developmental trends, excitatory and inhibitory synaptic connectivity was broadly balanced across local networks during each postnatal period studied.

### Mossy cell development and formation of canonical trisynaptic circuits

The DG-CA3-CA1 trisynaptic circuit is considered a fundamental principle of neuronal organization in the hippocampus. Cortical information enters the DG from the entorhinal cortex. DG granule cells project to CA3 pyramidal neurons through mossy fibers (which also synapse on local mossy cells). CA3 pyramidal neurons send Shaffer collaterals that synapse on CA1 pyramidal neurons (as well as forming local connections within the CA3). CA1 neurons then send efferent connections back to the entorhinal cortex. Information flow through this circuit is typically considered to be unidirectional in the mature hippocampus.

However, exceptions to this unidirectional flow, particularly in the immature hippocampus, have been documented. In a previous study we found that network activity propagated bidirectionally through this circuit (Shi et al., 2014). Activity that originated in the distal CA3 propagated toward the DG and CA1 at the same time in the young hippocampus (in P1–P14 mice). This propagation was mediated by AMPA receptors, and was developmentally transient, gradually disappearing by P14.

It is not clear how this bidirectional propagation develops, nor how the flow of activity becomes predominantly unidirectional in the mature hippocampal circuit. In past studies, biocytin staining and paired recording have shown that axon collaterals of CA3 pyramidal neurons directly innervate and activate mossy cells and GABAergic neurons (Scharfman, 1994) in the dentate and hilus. These neurons in turn sends axons to the molecular layer targeting granule cells. In the present study, we discovered that mossy cells received a relatively strong excitatory backprojection from the CA3 at P6–P7. The strength of this input declined at older ages. While inhibitory input from CA3 also decreased over the course of development, the latency to evoke inhibitory responses was faster than that for excitatory responses. Rapid onset of inhibition in mossy cells could prevent these neurons from activating granule cells, contributing to a unidirectional flow of information through the mature hippocampal circuit.

In addition to their potential roles in cognitive function mediated by the DG, hilar mossy cells are interesting due to their vulnerability to excitotoxicity in temporal lobe epilepsy (Scharfman and Myers, 2012). Mossy cell loss has been observed both in humans with temporal lobe epilepsy and in animal epilepsy models. The massive excitatory inputs from both the DG and the CA3 could put mossy cells at risk of cell death during epileptic firing, when levels of inhibitory input are chronically decreased. Indeed, in slice and *in vivo* recordings, we and others have observed that mossy cells receive a great deal of excitatory drive from local circuits via a continuous barrage of large spontaneous EPSPs (Scharfman and Schwartzkroin, 1988; Scharfman, 1993; Buckmaster and Schwartzkroin, 1995; Strowbridge and Schwartzkroin, 1996). Our present results suggest that robust excitatory drive from the DG in the mature hippocampus may provide the afferent inputs that lead to excitotoxicity.

## Synthesis

Reviewing Editor: Katalin Toth, Universite Laval Decisions are customarily a result of the Reviewing Editor and the peer reviewers coming together and discussing their recommendations until a consensus is reached. When revisions are invited, a fact-based synthesis statement explaining their decision and outlining what is needed to prepare a revision will be listed below. The following reviewer(s) agreed to reveal their identity: NONE.

Both reviewers agree that the current study contains novel and important information about the synaptic development of mossy cells. The study is generally carefully designed and executed. The most important aspect of the study is the dataset it provides on synaptic inputs from various developmental stages. Both reviewers made several points that aim to increase the quality of these datasets by providing additional information or precision on various values. The authors can address these points without new experiments. Reviewers' comments are highly constructive and answering these questions will significantly improve the manuscript.

Rev. #1.

The study presents an early developmental characterization of regional excitatory and inhibitory synaptic inputs to hippocampal dentate mossy cells. The authors use glutamate uncaging by laser scanning photostimulation in the granule cell layer, CA3 and hilus combined with whole cell recordings from presumed mossy cells (GAD negative neurons) at postnatal days 6-7, 13-14 and 21-28. They find a developmental shift in innervation with glutamatergic CA3 inputs to mossy cells dominating in young mice (P6-7) and dentate inputs being more frequent in older mice (P13-14 and P21-28). In parallel, they find that inhibitory connectivity to mossy cells is weak at 6-7 days with increase in dentate and hilar inhibitory inputs with development. The early developmental profile of mossy cell inputs characterized here is interesting. However basic methodological and analytical details need to be clarified before one can assess the implications of the study to circuit development.

Specific Comments:

1) Since putative mossy cells were patched based on GAD negativity, the exclusion/inclusion criteria need to be more rigorously defined. Were all mossy cells analyzed identified based on morphology? In results section, line 164-165, it is reported that mossy cell identification was confirmed by spiking patterns. However, using Cs to record inhibitory currents would alter firing properties. Overall, the inclusion criteria need to be better defined.

2) Were there any differences in development of mossy cell inputs along the dorso-ventral axis of the hippocampus?

3) It would help to include summary plots of mossy cell intrinsic physiological differences during development.

4) The authors use ‘summed’ inputs of as the primary output measure. This is however not defined in the manuscript. Summed amplitude (in pA) may be less relevant than Charge transfer when considering input strength. Also, is the summation spatial or temporal? If spatial, is there a rationale for linear summation versus one weighted for distance from recorded cell? It is also unclear how summed inputs relate to two critical parameters: a. number of inputs and b. strength of individual inputs. How does the summed input relate to the number of connected locations. What is being ‘summed’ needs to be defined.

5) In this regard, it would be useful to quantify the distance of the recorded cells from the DGC and CA3 to confirm that there were no systematic differences between age groups.

6) A ratio of number of input locations activated in DG vs. CA3, would be of relevance to assess connectivity

7) The relevance of event frequency is unclear- is this just a reflection of local circuit activity within the stimulated regions? The physical relevance of the various output parameters assessed needs to be discussed.

8) Please discuss the relevance of morphological changes with development and the changes in intrinsic physiology and excitatory inputs. The change in spine characteristics is of relevance to both.

9) Figure 4: include scale bar for images

Rev.#2.

In general, this is an interesting manuscript with nicely presented novel data. However, I think the manuscript can be improved by showing additional methodological details and clarifying some aspects of the interpretation of the data.

Suggestions:

The Introduction is clearly written in general. However, it should be also mentioned there what are the interesting stages of the hippocampal circuit and cellular developments, and which of these stages are represented by the current experimental animals. For example, the place field representation develops beyond the investigated age in other regions of the hippocampus (see Langston et al. 2010).

The Methods states that recordings were made “with good access resistance (usually &lt; 20 MΩ)”. However, the Rs values in Table 1 suggest higher access resistance during the recordings as the average values were around 30 MΩ. Please correct the above statement.

It is not clear whether the Rm and input resistance represent different properties of the recorded mossy cells. Please specify in the methods how these values were measured, including the membrane potential from which they were measured. If Rm and Rin values report similar properties deriving from current- and voltage-clamp measurements, please discuss why they were different in P13-14 and P21-28.

Results: The traces from P14 example cell in Fig.2. suggest that there are substantial differences in the firing properties at this age. For example, this cell lacked a burst like initial firing and the peak of the APs gradually decreased, unlike the younger and older example cells. If these cells are representative of the firing properties of each age group, further discussion and analysis would be needed.

There are large differences in the spike amplitudes when they were evoked with different current amplitudes (eg. Fig1.D). Because these unusual differences are the most obvious in the youngest cells (and consequently with the smallest Cm and smallest Cm/Cpip ratio), it raises the possibility that pipette capacitance was not correctly neutralized and consequently the bridge balance was improperly adjusted. These instrumental errors can cause the apparent differences in the AP amplitude and threshold. Therefore, please specify these compensation conditions and avoid drawing any conclusions about the spiking properties of the three age groups (eg. Line 182-183).

It is not obvious why the P6-7 mossy cells have dendrites in the granule cell layer, whereas the few days older cells avoid this region. This is what the sentence at lines 185-187 suggests. Please discuss this finding in the light of previous studies or revise the sentence to more precisely reflect the observations.

It is not clear why a 10 ms-long window was chosen for the exclusion of direct dendritic glutamate receptor activation. Activation of these receptors should occur within hundreds of microseconds after géutamate is released. Please justify this time window. Additionally, please provide a discussion about the possibility of masking of the aimed synaptic responses by direct glutamate responses. The notion is that synaptic responses may accur within 10 ms after the stimulus, especially from proximal neurons and fast responding cells (such as local interneurons).

Did the authors notice any differences in the excitability of the different presynaptic cells when they investigated photostimulation-evoked spikes (lines 217-221)? This information is important for understanding whether different excitability of the presynaptic sources may be accounted for differences in the number and strength of inputs. For example, there is a well known developmental changes in the excitability of granule cells, and the data in this manuscript also showed that the excitability of mossy cells develops during the investigated period.

It would be helpful if the analyzed data categories are indicated on the graphs, for example ‘strength’ on Fig.5A and ‘number of events’ Fig.5B. It would help understanding the IPSC-graphs similarly in Fig6. Furthermore, it is not indicated whether these graphs show individual slices or pooled data.

typos: p.3. “Mossy cells are the principal excitatory neurons...” is more correct if said as “Mossy cells are principal excitatory neurons...”

Line212. “ We current-clamp neurons...”

Line 229. “received a majority of”
